# The Treatment of Whitlow

**Published:** 1909-08-21

**Authors:** 


### Surgery.

### THE TREATMENT OF WHITLOW.

Many varieties of whitlow are met with, and they are sub-divided according to the anatomical layer in which the infection starts. The least serious is the subcuticular variety, in which an abscess forms under the skin itself. This is generally found in the terminal phalanx as the result of infection of a superficial wound or abrasion; it forms a raised, dead-white, or yellow, fluctuating swelling, which is tender, but does not throb. The treatment consists of incising the swelling at an early moment; some surgeons cut away all the skin which has been raised from the subcutaneous tissue to the extreme limit of the abscess. This is not a good proceeding. The dead skin forms an admirable protective covering for the damaged area, while the new epidermis is being formed underneath, and it can easily be removed later, when the process of regeneration is near completion. But if this skin is removed early, a new area exuding serum is exposed, which sticks to the dressings, and each time these are removed the healing process is retarded.

A subcutaneous whitlow is a much more serious affair. It results from a prick or stab with a sharp but septic implement. A rusty nail is responsible for a great number of them. This is also usually found in the terminal phalanx since that is the portion of the finger most liable to injury. The whole of the subcutaneous tissues of the pulp of the finger become acutely inflamed. The end of the finger is enlarged, the skin is shiny and red, and excessively tender if touched. This variety is excruciatingly painful, a fact which is due to the intimate way in which the skin is held down to the subcutaneous tissues in this region. The pus is therefore retained under tension, a circumstance which is always associated with great pain. The pain is increased by anything which causes engorgement of the vessels of the finger, such as keeping the arm in a dependent position or the increased warmth of bed. The patient often complains of an intense throbbing at night, which prevents him from sleeping at all. It is quite astonishing to what a great extent the general health of the patient may be affected by an apparently insignificant focus. But the constant pain and the insomnia are capable of affecting the patient to a serious degree unless relief is afforded by early surgical interference. Further than this, the infection spreads along the lymphatics of the arm, so that these become visible as angry-looking pink streaks, and the epitrochlear and axillary glands may become enlarged, and finally suppurate.

The obvious duty of the surgeon in such a case is to allow free exit to the toxins by incision. The mistake that is usually made is that an insufficient incision is made in the first place in the hope that the infection will subside. If possible, a general anæsthetic should always be given (gas is generally sufficient), and an incision should be made which is not only free but ensures adequate drainage. An excellent way of doing this is to make two slightly elliptical incisions and to remove entirely the intervening portion of skin. This will effectually prevent the edges of the wound from falling together and so locking up the pus again. Fomentations should, of course, be applied. The writer prefers to use gauze for this rather than the lint which is usually employed. Even then a second, or possibly a third, operation may be necessary.

Occasionally the infection travels deeply towards the phalanx, and periostitis and partial necrosis of the bone itself occurs. An incision must then be made right down to the bone, and the necrosed portions be removed by scraping. When this occurs the terminal joint of the finger often looks so completely disorganised that amputation appears to be necessary. This should never be done with undue haste. The ultimate result of treatment by adequate and continued drainage is nearly always successful, unless the entire phalanx has necrosed, and the deformity left is surprisingly little.

A very troublesome form of whitlow is the perionychial. Here the infection starts at the base of the nail, where the skin is particularly liable to abrasion. An abscess forms round the base of the nail and underneath it, and the nail itself dies wholly or in part. But here, again, the trouble is mainly due to inefficient treatment at the outset. It must be recognised that the nail will die, and it is better to remove it completely than to allow it to slough off of its own accord. There are two distinct advantages in doing this—the new nail is less likely to be deformed, and free and immediate drainage is given to the abscess. If granulations have already formed on the matrix, these may be scraped and touched with silver nitrate.

Sometimes the infection invades the tendon sheath (thecal whitlow), and the whole finger is swollen as far down as the metacarpo-phalangeal joint. But a subcutaneous whitlow may also spread down the whole finger, and great care is therefore required in operating not to convert what is only a subcutaneous whitlow into a thecal one by opening the sheath. But if the sheath is already involved it must be laid freely open, since it is only by doing this that the life of the tendon can be saved. The prognosis is, however, never good; in more than half the cases one or other of the flexor tendons dies and sloughs away, leading to great impairment of the use of the finger. A thecal whitlow in the little finger or thumb is more serious than in the other three, because the tendon-sheath is continuous in these digits with the palmar bursa, which thus may become infected.

It should be added that the general health of the patient should be attended to. A tonic composed of quinine and iron is of benefit, and the bowels should be kept freely open. It is also advisable to give a vaccine made from the organism found in the pus, which must be cultivated for this purpose. There can be little question that this treatment increases the patient's resistance to the infection and helps him to overcome it.

